# Domain shuffling of a highly mutable ligand‐binding fold drives adhesin generation across the bacterial kingdom

**DOI:** 10.1002/prot.26487

**Published:** 2023-03-20

**Authors:** Rob Barringer, Alice E. Parnell, Aleix Lafita, Vivian Monzon, Catherine R. Back, Mariusz Madej, Jan Potempa, Angela H. Nobbs, Steven G. Burston, Alex Bateman, Paul R. Race

**Affiliations:** ^1^ School of Biochemistry University of Bristol, University Walk Bristol BS8 1TD UK; ^2^ BrisSynBio Synthetic Biology Research Centre University of Bristol, Life Sciences Building Tyndall Avenue Bristol BS8 1TQ UK; ^3^ European Molecular Biology Laboratory European Bioinformatics Institute (EMBL‐EBI) Wellcome Genome Campus Hinxton CB10 1SD UK; ^4^ Department of Microbiology, Faculty of Biochemistry, Biophysics, and Biotechnology Jagiellonian University Krakow Poland; ^5^ Department of Oral Immunology and Infectious Diseases University of Louisville School of Dentistry Louisville Kentucky USA; ^6^ Bristol Dental School, University of Bristol Lower Maudlin Street Bristol BS1 2LY UK

**Keywords:** adhesin, bioinformatics, fibril, *Streptococcus gordonii*, X‐ray crystallography

## Abstract

Bacterial fibrillar adhesins are specialized extracellular polypeptides that promote the attachment of bacteria to the surfaces of other cells or materials. Adhesin‐mediated interactions are critical for the establishment and persistence of stable bacterial populations within diverse environmental niches and are important determinants of virulence. The fibronectin (Fn)‐binding fibrillar adhesin CshA, and its paralogue CshB, play important roles in host colonization by the oral commensal and opportunistic pathogen *Streptococcus gordonii*. As paralogues are often catalysts for functional diversification, we have probed the early stages of structural and functional divergence in Csh proteins by determining the X‐ray crystal structure of the CshB adhesive domain NR2 and characterizing its Fn‐binding properties in vitro. Despite sharing a common fold, CshB_NR2 displays an ~1.7‐fold reduction in Fn‐binding affinity relative to CshA_NR2. This correlates with reduced electrostatic charge in the Fn‐binding cleft. Complementary bioinformatic studies reveal that homologues of CshA/B_NR2 domains are widely distributed in both Gram‐positive and Gram‐negative bacteria, where they are found housed within functionally cryptic multi‐domain polypeptides. Our findings are consistent with the classification of Csh adhesins and their relatives as members of the recently defined polymer adhesin domain (PAD) family of bacterial proteins.

## 1 INTRODUCTION

Many bacteria present filamentous polypeptides on their surfaces termed adhesins. These extracellular proteins enable binding to biotic and abiotic surfaces, often via an intimate and highly specific interaction with a partner ligand or receptor.^1^ Adhesin‐mediated interactions make important contributions to colonization and pathogenicity,^2^ with elucidation of the structures and functions of these proteins considered critical for informing our fundamental understanding of the mechanisms of microbial adhesion, and in directing future efforts in the development of antiadhesin‐based therapies or antimicrobial surface coatings.^3^

Most bacterial adhesins possess an equivalent gross architecture comprising a cell wall tethered elongated stalk with an adhesive tip. These polypeptides are generally classified as belonging to one of two distinct groups; pili or fibrils.^1^ Pili are homomeric assemblies typically composed of covalently or noncovalently linked precursor proteins.^4^ In contrast, fibrillar adhesins usually comprise a single polypeptide chain.^2^ Fibrillar adhesins have been found widely across the bacterial tree of life^5^ and vary significantly in their molecular architectures and ligand‐binding specificities. Despite their diversity, subsets of adhesive domains from fibrillar adhesins exhibit some degree of identity. For example, the adhesive domains of the Ag I/II‐like polypeptides SspB, SpaP, PrgB, and GbpC, which constitute the recently designated polymer adhesin domain (PAD) superfamily,^6^ or those from the adhesins Sgo0707 and CshA, which exhibit significant structural identity despite their disparate amino acid sequences.^7^, ^8^

**FIGURE 1:**
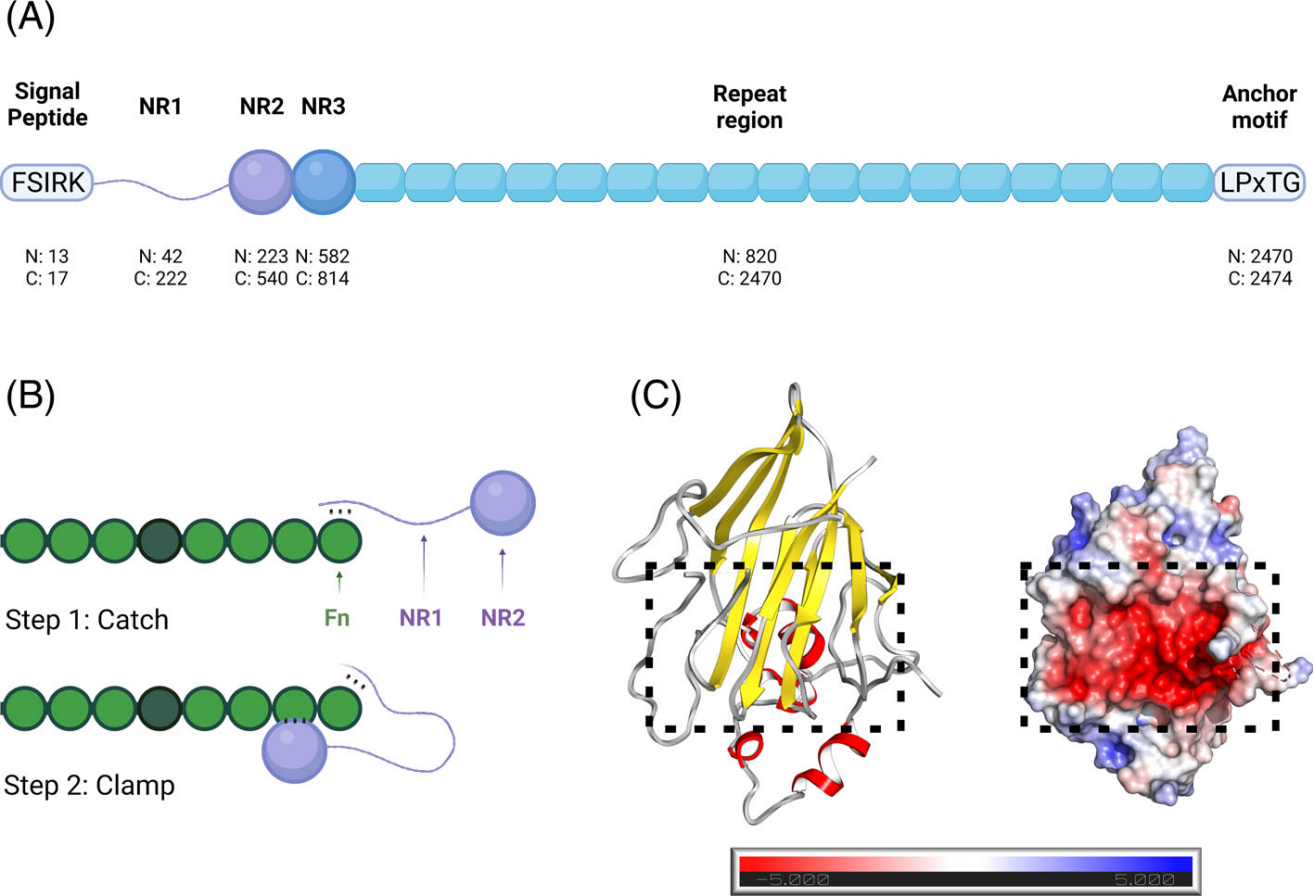
Structure and function of CshA. (A) Domain architecture of CshA, which comprises from N‐terminus to C‐terminus, an “FSIRK” sorting signal, the nonrepeat domains NR1, NR2, and NR3, 17 repeat domains, and an LPxTG anchoring motif. N‐ and C‐terminal domain boundaries are indicated (sequence positions designated “N” and “C”). (B) Illustration of the “catch‐clamp” mechanism of Fn binding by the CshA_NR1/NR2 domains. Fn is depicted in green, CshA domains are in purple. It is unknown which of the Fn domains are targeted by CshA, thus this illustration represents a simplified depiction. (C) Ribbon representation (left) and electrostatic surface potential (right; red, negative; blue, positive; white, neutral) of the CshA_NR2 domain. The Poisson‐Boltzmann electrostatic scale bar shown depicts a potential of ± 5 kT/e (red to blue, respectively). The Fn‐binding site of CshA_NR2 is indicated by a dotted box.

The *Streptococcus gordonii* (*S. gordonii*) fibrillar adhesin *c*ell *s*urface *h*ydrophobicity protein *A* (CshA) is an ∼259 kDa cell wall anchored polypeptide that confers fibronectin (Fn)‐binding properties to this bacterium.^9^, ^10^ CshA comprises an N‐terminal “nonrepeat” (NR) region, which houses the adhesive CshA_NR2 domain, fused to a stalk‐forming “repeat region” (RR), incorporating 17 sequentially arrayed ~100 amino acid domains (Figure 1A).^11^ In previous studies of the CshA non‐repeat region, we demonstrated that Fn binding by CshA occurs via a distinctive “catch‐clamp” mechanism, where the intrinsically disordered NR1 domain of the protein functions to “catch” Fn, through the formation of a rapidly assembled but readily dissociable precomplex, enabling its neighboring NR2 domain to form a tight‐binding interaction with Fn “clamping” the two polypeptides together (Figure 1B).^8^ Subsequent elucidation of the crystal structure of CshA_NR2 revealed a distinctive β‐sandwich core fold, composed of two antiparallel β sheets decorated by α‐helices. These studies also demonstrated that CshA_NR2 possesses a highly negatively charged pocket on its surface consistent with a role in Fn binding (Figure 1C). Complementary studies demonstrated that CshA_NR2 binds tightly to Fn in vitro, even in the absence of CshA_NR1, confirming its implicit role in Fn binding.

Here we expand the scope of our studies of Csh proteins by focusing on the *S. gordonii* CshA paralogue CshB. This polypeptide is antigenically related to CshA, but is encoded for by a gene resident at a distant chromosomal locus.^11^ CshB has previously been shown to play a role in Fn binding by *S. gordonii*, with Δ*csh*A strains retaining some Fn‐binding capability as compared to a Δ*csh*A/Δ*csh*B double mutant that does not adhere to Fn‐decorated surfaces.^11^ In addition, deletion of either of these genes has been shown to abrogate colonization of the murine oral cavity by *S. gordonii*.^10^

In this study, we have used in vitro binding assays of WT and mutant CshA polypeptides to unambiguously identify the Fn‐binding pocket of CshA_NR2. Building on these findings we have elucidated the X‐ray crystal structure of CshB_NR2, which is found to possess a core fold analogous to that of CshA_NR2. Inspection of the ligand‐binding pocket of CshB_NR2 reveals significant variations in the amino acid composition of this region of the polypeptide as compared to CshA_NR2. Complementary in vitro binding studies demonstrate that these changes translate into a reduced Fn‐binding affinity. To investigate the generality of our findings, a bioinformatics analysis was undertaken in an effort to identify more distantly related homologues of CshA/B_NR2 and assess their distribution in bacterial adhesins. This analysis reveals an unexpectedly broad distribution of CshA/B_NR2‐like adhesive domains in both Gram‐positive and Gram‐negative fibrillar adhesins. These polypeptides appear to arise as a consequence of domain shuffling, yielding novel modular architectures of variable lengths.^5^, ^6^, ^12^ Our analyses also reveal that well‐characterized structural homologues of CshA/B_NR2 possess an equivalent β‐sandwich core fold but vary in binding cleft architecture and partner selectivity. These findings are consistent with an evolutionary diversification of function, originating from a highly mutable progenitor fold. In undertaking this analysis, we also identify the adhesive domains of Sgo0707 and CshA/B as members of the previously reported PAD family of bacterial polypeptides^6^ and demonstrate that domain shuffling within PAD family members functions as a mechanism for the generation of adhesins in both Gram‐positive and Gram‐negative species.

## 2 EXPERIMENTAL PROCEDURES

### 2.1 Gene cloning and protein production

Recombinant CshA_NR2 was expressed and purified as described previously.^8^ For CshB_NR2, a DNA fragment corresponding to residues 211–528 of the full‐length CshB polypeptide was PCR‐amplified from *S. gordonii* DL1 chromosomal DNA using CloneAmp™ HiFi PCR premix (Takara Bio), employing appropriate primers incorporating consensus sequences to enable cloning into the expression vectors pOPINE or pOPINF (Table S1).^13^ Resulting PCR products were ligated into either pOPINE, pre‐cut with *Pme*l and *Nco*I, or pOPINF, pre‐cut with *Kpn*I and *Hind*III, using the In‐Fusion™ system (Takara Bio). The resulting plasmids, termed *csh*B_NR2::pOPINE and *csh*B_NR2::pOPINF, encode a C‐terminally hexa‐histidine‐tagged variant of CshB_NR2 and an N‐terminally hexa‐histidine‐tagged variant of CshB_NR2, respectively. Protein produced using *csh*B_NR2::pOPINE was used for crystallization studies, with protein produced using *csh*B_NR2::pOPINF employed in binding studies. For the CshA_NR2 single mutants D365W, E367W, M481W and the CshA_NR2 triple mutant D365W/E367W/M481W, codon optimized synthetic genes were sourced from the GeneArt service of Thermo Fisher. Synthetic genes were amplified by PCR using appropriate primers (Table S1) and ligated into the vector pOPINF as described above for *csh*B. All constructs were verified by DNA sequencing.

For the expression of recombinant CshB_NR2 and each of the four CshA_NR2 mutants, *E. coli* BL21 (DE3) cells harboring *csh*B_NR2::pOPINE, *csh*B_NR2::pOPINF, *csh*A_NR2_ D365W::pOPINF, *csh*A_NR2_E367W::pOPINF, *csh*A_NR2_M481W::pOPINF, or *csh*A_NR2_ D365W/E367W/M481W::pOPINF, were grown in 1 L of LB broth supplemented with carbenicillin (100 μg/mL) to an OD_600nm_ = 0.4–0.6. Protein expression was induced by the addition of IPTG to a final concentration of 1 mM, followed by further incubation for 16 h at 18°C with shaking. Bacteria were harvested by centrifugation, supernatants discarded, and resulting cell pellets frozen at −80°C. For protein purification, cell pellets were resuspended in either 40 mM Tris–HCl, 500 mM NaCl, pH 7.5 (CshB_NR2 used for crystallization studies) or 637 mM NaCl, 2.7 mM KCl, 10 mM Na_2_HPO_4_, 1.8 mM KH_2_PO_4_, pH 7.4 (CshA_NR2 WT and mutants, and CshB_NR2 used for Microscale Thermophoresis experiments), and lysed with a cell disrupter (Constant Systems Ltd.) at 25 000 p.s.i. Cell lysates were clarified by centrifugation and supernatants applied to a 5 mL Hi‐Trap chelating column preloaded with Nickel (Cytiva). Recombinant proteins were eluted using an imidazole gradient of 20–500 mM over a volume of 60 mL at 1 mL min^−1^. Fractions containing proteins of interest were pooled, concentrated, and further purified by passage through a Hi‐Load 16/600 Superdex 75 column (GE Healthcare) pre‐equilibrated in either 40 mM Tris–HCl, 500 mM NaCl, pH 7.5 (CshB_NR2 used for crystallization studies), or 637 mM NaCl, 2.7 mM KCl, 10 mM Na_2_HPO_4_, 1.8 mM KH_2_PO_4_, pH 7.4 (CshA_NR2 WT and mutants, and CshB_NR2 used for Microscale Thermophoresis experiments). All recombinant proteins were subjected to MALDI MS analysis following trypsin digestion to confirm their identity.

### 2.2 Assessment of protein secondary structure by circular dichroism spectroscopy

Circular dichroism (CD) spectra of WT and mutant CshA_NR2 polypeptides were collected using a Jasco J‐1500 spectrophotometer fitted with a Peltier temperature control unit. Proteins were dialysed overnight at 4°C into buffer comprising 10 mM sodium phosphate, 100 mM sodium fluoride, pH 7.4, prior to analysis. CD spectra were collected from 300 μL samples of CshA_NR2 WT, CshA_NR2 D365W, CshA_NR2 E367W, CshA_NR2 M481W and CshA_NR2 D365W/E367W/M481W, each at 1.0 mg/mL, at 4°C, using a 1 mm path length cuvette (Hellma Analytics) pre‐purged with nitrogen gas to cleanse any contaminants prior to sample analysis. For all experiments, a high‐tension (HT) voltage of <700 V was taken as the quality threshold for the CD signal. Final spectra were generated as averages of four repeat scans, with appropriate protein‐free buffer spectra subtracted.

### 2.3 CshB_NR2 crystallization and structure elucidation

Conditions supporting the growth of CshB_NR2 crystals were initially identified using the sitting drop method of vapor diffusion at 20°C, employing commercially available screens (Molecular Dimensions Ltd.). Diffraction‐quality crystals were grown in 0.1 M SPG buffer (succinic acid, sodium dihydrogen phosphate and glycine, in the molar ratio 2:7:7) pH 5, 25% w/v PEG 1500. Crystals selected for diffraction data collection were mounted in appropriately sized LithoLoops (Molecular Dimensions) and flash‐frozen in liquid nitrogen without the addition of extraneous cryoprotectant. Diffraction data were collected at Diamond Light Source, UK, on beamline I03. Data were processed with xia2^14^ and DIALS^15^ and the CshB_NR2 structure was determined by molecular replacement using PHASER,^16^ employing the CshA_NR2 structure (5L2D) as a search model. The initial CshB_NR2 model was subjected to iterative rounds of model building (COOT^17^) and refinement (REFMAC^18^) undertaken using the CCP4i2 software suite.^19^, ^20^ The structure of CshB_NR2 has been deposited in the PDB^21^ with the accession code 6YZG. Structural figures have been prepared using PyMoL.^22^ Electrostatic surface analyses have been performed using the APBS plugin^23^ in PyMol following molecule preparation with pdb2pqr using PropKa^24^ applying an AMBER 99 force field. APBS was implemented with a grid spacing of 0.50 and a surface visualization of the Poisson‐Boltzmann electrostatic potential of ±5 kT/e.

### 2.4 In vitro Fn‐binding studies

In vitro quantitation of cellular fibronectin (cFn) binding by WT and mutant CshA_NR2 polypeptides, and WT CshB_NR2, was performed using Microscale Thermophoresis (MST). Prior to analysis commercially sourced cFn (Sigma) was labeled with RED‐NHS dye (NanoTemper Technologies) using the Monolith Protein Labelling kit (NanoTemper Technologies) as per the manufacturer's instructions. Labeled cFn (50 nM) was mixed with 16 concentrations (92 pM–3 μM) of monomeric WT or mutant CshA_NR2, or WT CshB_NR2, in buffer comprising 637 mM NaCl, 2.7 mM KCl, 10 mM Na_2_HPO_4_, 1.8 mM KH_2_PO_4_, pH 7.4. The resulting samples were analyzed using a Monolith NT.115 (NanoTemper Technologies). MST power was set to 40%, with 20% excitation power, and a temperature constraint of 22–23°C. Validation of capillary scans was performed prior to sample analysis, with no indication of adsorption and with comparable fluorescence signals. Bovine Serum Albumin (BSA) was used as a negative control and showed no evidence of binding to cFn. All measurements were performed in triplicate. MST traces and binding data were analyzed using the MO. Control and MO. Affinity software.

### 2.5 CshA/B_NR2 sequence conservation analysis

For sequence conservation analysis, homologues of CshA_NR2 were identified using PSI‐BLAST^25^ searching against nonredundant protein sequences in the organism category of *Streptococcaceae* (taxid:1300) in NCBI.^26^ The search was limited to the generation of 500 matches with an *E*‐value cut‐off of 0.001. Searches applied a word size of 3, a BLOSUM62 matrix and an existence/extension gap cost of 11/1, alongside the default conditional compositional score matrix adjustment option and a PSI‐BLAST threshold of 0.005. A total of five iterations were performed generating the final count of 500 sequences, which included CshB_NR2. To assess the degree of conservation, the sequences were aligned using MAFFT 7.471^27^ and visualized using the CLC Genomics Workbench 12 software suite (CLCGW12, https://digitalinsights.qiagen.com). Residues showing >95% conservation were manually mapped onto the CshA_NR2 crystal structure.

### 2.6 CshA/B_NR2 sequence homologue acquisition, alignment, and phylogenetic analysis

For phylogenetic analyses sequences of CshA_NR2 homologues, including those of distant relatives, were identified by performing 10 iterative PSI‐BLAST searches executed with no restriction on the number of sequences returned and without taxon exclusions, applying the same parameters as outlined above. This approach returned 2056 sequence matches. Hits were aligned using MAFFT 7.471 and constructed into a phylogenetic tree using rapidnj^28^ with default parameters, and trees were subsequently visualized in CLCGW12. Sequences from resultant clusters were manually picked (200 hits total) for full architecture annotation using InterPro.^29^ Batch Entrez^30^ was used to retrieve sequences of full‐length CshA homologues (1804 sequences returned, 252 rejected) with low‐to‐moderate sequence identity to the polypeptide. Candidates were triaged using sequence alignments generated using MUSCLE 3.8.31,^31^ as implemented in the EMBL‐EBI API^32^ with default parameters. Clusters found to contain polypeptides housing adhesin associated domains were color coded using CLCGW12 in a circular cladogram and annotated using GIMP.^33^ Following annotation with InterPro, a subset of sequences were selected and visualized using Biorender^34^ in an effort to establish the diversity of domain architectures.

To identify structural homologues of CshA/B_NR2, searches were conducted using the DALI web server^35^ employing the monomeric forms of CshA_NR2 and CshB_NR2 as search models. Known adhesive domains with a DALI *Z* score of >6.0 were selected for comparative structural analysis. MUSCLE was used as before to produce a sequence identity matrix.

### 2.7 CshA_NR3 domain boundary reannotation

To unambiguously establish the C‐terminal domain boundary of the previously assigned CshA_NR3 region, archetype CDD^36^ consensus sequences of NR3 domains and their associated downstream regions of sequence in the 2056 PSI‐BLAST library were aligned against the sequence of full‐length CshA using EMBOSS Needle.^32^ Alignments with a Needle score > 100.0 were used to predict the annex sites of NR3. Based on these analyses the two domains constituting the NR3 region of CshA and its relatives were subsequently termed the ‘GEVED’ domain, and CshA repeat region 1 (RR1).

### 2.8 Bioinformatic characterization of CshA/B_NR2 containing polypeptides

The 2056 homologues of CshA_NR2 identified using an iterative PSI‐BLAST search were aligned using MUSCLE and an NR2 hidden Markov model (HMM) was created using the HMMER tool (hmmer.org, version 3.1b2). Both steps were conducted with default options. The full‐length sequences retrieved from Batch Entrez were run against the NR2 HMM and the Pfam HMM database (version 33.1),^37^ which included the newly defined GEVED domain (Pfam:PF20009) using the HMMER tool (version 3.1b2) with the gathering (GA) threshold or the domain threshold (−domE 0.1) option, respectively. The results were combined and overlapping domain annotations were excluded based on the hmmsearch annotation score. To investigate which known stalk domains are used in combination with the NR2 domain in the full‐length sequences, the Monzon et al. HMM collection of known stalk domains^5^ and the GEVED stalk domain were counted per phyla, where stalk domains from the same domain family were only counted once per protein, and a heatmap was created using seaborn.^38^ Subsequent analyses required a sample library of full‐length proteins, which was generated by identifying protein sequences with an N‐terminal signal peptide and a C‐terminal anchoring domain. To this end, proteins with anchor domains were identified based on the known anchor domain collection from Monzon et al. and the OmpA (Pfam: PF00691) anchor domain.^5^ Additionally, regular expression was used to search within the last 50 C‐terminal residues of the full‐length sequences for sortase motifs (“LPxT[G|A|N|D],” “NPxTG,” “LAxTG,” “NPQTN,” “LPxGA,” “IPxTG,” whereby “x” can be any amino acid). The TMHMM webserver^39^ (https://services.healthtech.dtu.dk/service.php?TMHMM-2.0, version 2) was used to search for transmembrane helices for sequences without a detected anchor. Sequences in which only one transmembrane helix was found that was also marked as a possible signal peptide were excluded in further analyses as they did not represent anchor domains. Proteins with signal peptides (YSIRK_signal (Pfam:PF04650), Planc_extracel (Pfam:PF07595), TAT_signal (Pfam:PF10518), ESPR (Pfam:PF13018)) were identified in the combined HMMER search output file, and the search was further extended by using the SignalP webserver (version 5.0, https://services.healthtech.dtu.dk/service.php?SignalP-5.0).^40^ The distribution of signal peptides and anchor domains between the four main phyla (Firmicutes, Actinobacteria, Proteobacteria, Bacteroidetes) was subsequently plotted using bokeh.^41^ A list of identifiers of full‐length sequences with a known anchor and signal peptide was subsequently created, generating a list of full‐length protein sequences that does not include partial sequences. To create the gradient heatmap the NR2 HMM was run against the full‐length sequences within the list, and the fractional position of the NR2 domain was calculated based on the length and envelope domain location information from the HMMER search output file. For each of the four main phyla the fractional NR2 locations were summed up at a percentage from 1 to 100 representing the distance from the N‐terminus, relative to the total length of the protein. The sum is normalized by the total number of sequences per phyla and the results were plotted using seaborn. For the subsequent length and domain analysis, the sequences were split into the main four phyla and “others,” representing less abundant phyla. The sequence lengths per phyla were plotted using seaborn.

### 2.9 Structure predictions for NR2‐like domains

Structure predictions for NR2‐like domains were performed using AlphaFold2 employing the Colabfold server.^42^ Selected sequences identified from the PSI‐BLAST pool of NR2‐like homologues were input into the server with default settings applied. These corresponded to: no template mode enabled, msa_mode: MMseqs2 (UniRef+Environmental), pair_mode: unpaired+paired, model_type: auto, num_recycles: 3. The server generated five .pdb models for each input sequence, with the highest ranking selected for further analysis.

## 3 RESULTS

### 3.1 Elucidation of the Fn‐binding pocket of CshA_NR2

**FIGURE 2:**
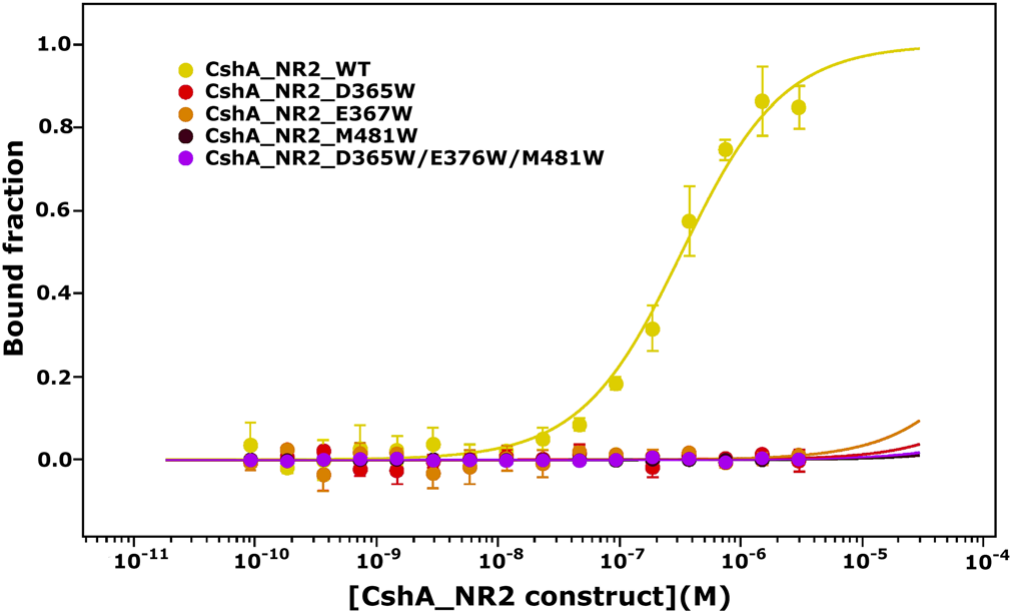
In vitro quantitation of cFn binding by WT and mutant CshA_NR2 polypeptides. MST traces of cFn binding by CshA_NR2 WT (yellow), CshA_NR2 D365W (red), CshA_NR2 E367W (orange), CshA_NR2 M481W (black), and CshA_NR2 D365W/E367W/M481W (pink). All experiments were conducted using cFn at a final concentration of 50 nM. Data have been fitted to a single‐site dose–response binding model. Error bars correspond to standard deviations from the mean calculate from three repeats of each measurement.

In previous studies, we identified CshA_NR2 (residues 223–540 of the full‐length polypeptide) as a high‐affinity Fn‐binding domain.^8^ Structural studies of this domain revealed a large negatively charged cleft on the surface of the polypeptide which was proposed to function as the site of Fn binding (Figure 1C).^8^ In an effort to establish the explicit role of this cleft in Fn binding by CshA_NR2, we undertook in vitro binding studies using Microscale Thermophoresis (MST), comparing the binding affinity of cFn to WT monomeric CshA_NR2 and selected CshA_NR2 mutants. Four CshA_NR2 mutants were generated; three single mutants (D365W, E367W, M481W) which incorporate tryptophan residues within the proposed Fn‐binding site of CshA_NR2, and a triple mutant (D365W/E367W/M481W) incorporating each of the three single point mutations. Far UV CD spectroscopy confirmed that all four mutant proteins were equally as well folded as WT CshA_NR2 (Figure S1). In addition, analysis by size exclusion chromatography demonstrated that each of the four mutant proteins exhibited hydrodynamic properties equivalent to those of the WT polypeptide (Figure S1). For MST studies, cFn was mixed with increasing concentrations of monomeric WT CshA_NR2 or each of the four mutants, to produce binding curves from which the equilibrium dissociation constant of each protein for cFn could be determined. Our MST binding studies revealed that in contrast to WT CshA_NR2, which was found to bind to cFn with a *K*_D_ value of 302 ± 44 nM, we observed no evidence of cFn binding by our CshA_NR2 mutants (Figure 2). Together, these data demonstrate that disruption of the highly charged surface cleft of CshA_NR2 through mutation abrogates cFn binding, thus establishing this region of the polypeptide as the Fn‐binding site of CshA_NR2. It is notable that the *K*_D_ value for cFn binding by CshA_NR2 is lower than that previously determined using Biolayer Interferometry (BLI; 500 nM),^8^ a disparity that may reflect the increased sensitivity of MST as compared to BLI.^43^

### 3.2 X‐ray crystal structure of CshB_NR2

To further advance our understanding of Csh adhesins, we next sought to elucidate the X‐ray crystal structure of the NR2 domain of the CshA paralogue CshB (residues 211–528 of the full‐length polypeptide). Although the two adhesins share 70% sequence identity, and CshB has been previously shown to contribute to Fn binding in *S. gordonii*,^10^ it was unclear if Fn binding was the primary role of the CshB_NR2 domain, as gene duplicated paralogues are frequently the starting point for functional diversification.^44^, ^45^

The DNA coding region for CshB_NR2 was PCR amplified from *S. gordonii* DL1 chromosomal DNA and cloned into the vector pOPINE, with the resulting plasmid used to facilitate the overexpression of a C‐terminally his‐tagged variant of CshB_NR2 in *E. coli*. Recombinant CshB_NR2 was purified to homogeneity using nickel affinity chromatography followed by size exclusion chromatography (SEC). Two distinct peaks were observed in the SEC chromatogram, with retention profiles consistent with monomeric and dimeric forms of CshB_NR2. Despite repeated efforts, only the dimeric form of CshB_NR2 proved amenable to crystallization. The structure of the CshB_NR2 dimer was subsequently determined to 1.4 Å resolution by molecular replacement using the CshA_NR2 structure as a search model (PDB code 5L2D).^8^ Data collection and refinement statistics for the CshB_NR2 structure are provided in Table 1.

**TABLE 1:** Summary of X‐ray data collection and refinement statistics.

CshB_NR2	
Data Collection	
Space group	*P*4_1_2_1_2
Unit cell dimensions (Å): A, B, C	100.390, 100.390, 63.302
Unit cell angles (°): *α*, *β*, *γ*	90, 90, 90
Crystal system	Tetragonal
Resolution (Å)	70.99–1.4 (1.4–1.4)
*R*_merge_	0.05 (0.552)
Number of reflections	63 721 (6210))
Multiplicity	6.7 (4.9)
Overall signal‐to‐noise ratio (I/*σ*)	15.24 (2.17)
CC_1/2_ (%)	0.998 (0.796)
Completeness (%)	100 (99.1)
Refinement	
Resolution (Å)	70.99–1.4 (1.4–1.4)^a^
*N* ^o^ reflections used in refinement	63 719 (6210)
*R*_work_	0.183
*R*_free_	0.195
*N* ^o^ protein atoms used in refinement	2160
*N* ^o^ water atoms used in refinement	284
*B* factors for protein atoms (Å^2^)	21.24
*B* factors for water atoms (Å^2^)	30.32
RMS deviations—length (Å)	0.017
RMS deviations—angle (°)	1.99
Ramachandran‐favored residues (%)	98.5
Ramachandran outlying residues (%)	0

*Note*: Values in parentheses represent the highest‐resolution shell.

**FIGURE 3:**
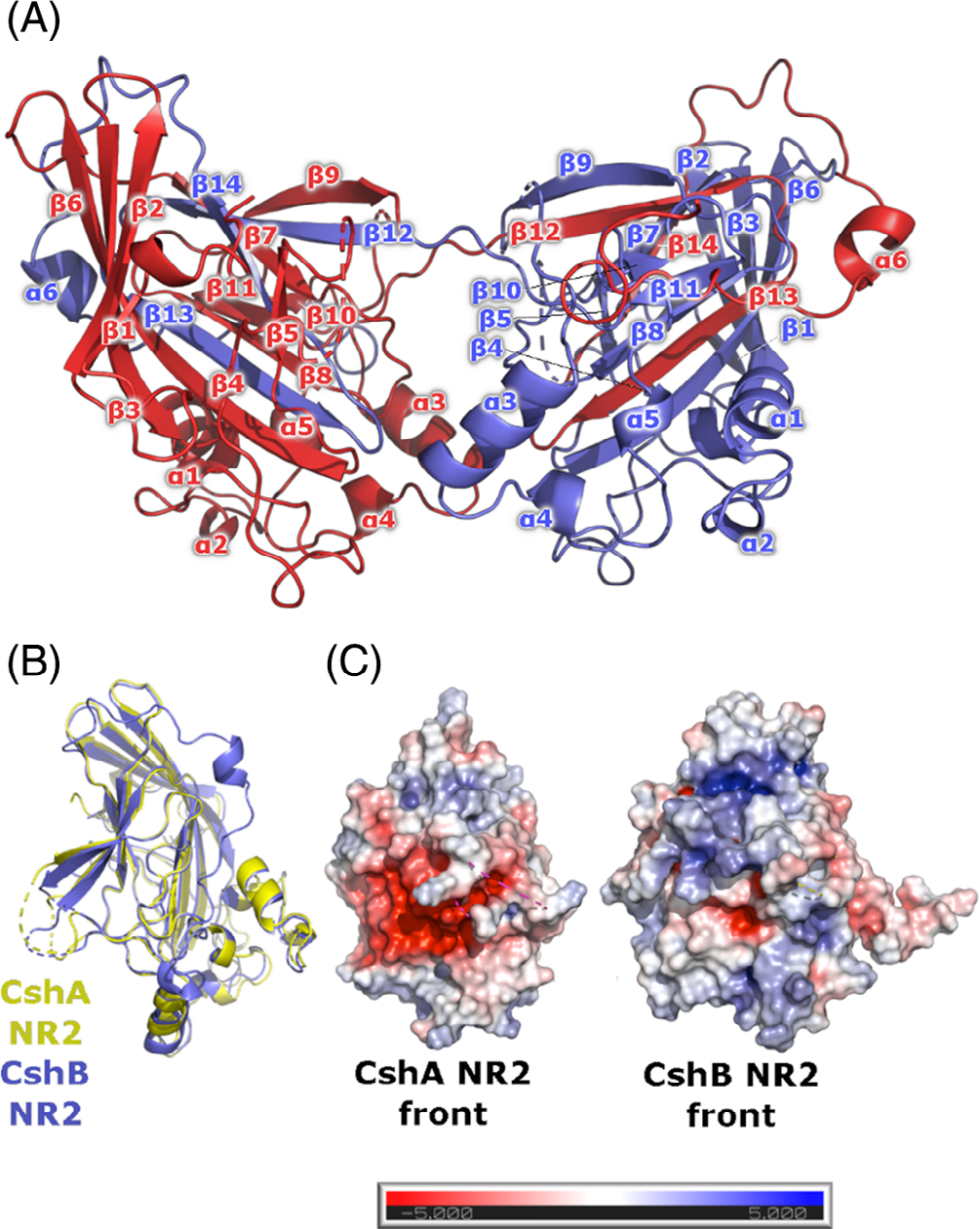
Crystal structure of CshB_NR2. (A) Structure of the strand‐swapped CshB_NR2 homodimer. Individual monomer chains are differentially colored (red and blue). (B) Superposition of the structures of monomeric CshA_NR2 and CshB_NR2. CshA_NR2 is shown in yellow, CshB_NR2 in blue. (C) Electrostatic surface potentials of monomeric CshA_NR2 and CshB_NR2. The Poisson–Boltzmann electrostatic scale bar indicates potentials of ± 5 kT/e (red to blue, respectively).

The crystal structure of CshB_NR2 (Figure 3A) reveals a strand‐swapped homodimer, wherein residues 442–528 of each monomer are buried in the central core of the associated dimer partner. The exchanged region comprises β‐strands 12–14 of each polypeptide chain. Using our CshB_NR2 strand‐swapped dimer structure as a template, a model of the biologically relevant monomeric form of the protein was generated and used for further analysis. Monomeric CshB_NR2 adopts an overall fold equivalent to that of CshA_NR2 (Figure 3B), with a Cα root‐mean‐square deviation (RMSD) between the two structures of 1.0 Å. The CshB_NR2 fold comprises a β‐sandwich core formed from two antiparallel β‐sheets, one of eight β‐strands and the other of six β‐strands. The interface of the two sheets is populated by hydrophobic residues. The periphery of the β‐sandwich is decorated with six α‐helices, which are located between strands β3/β4 (α1 and α2), β9/β10 (α3, α4, and α5) and after β14 (α6; Figure 3A). In contrast to previous crystallographic studies of CshA_NR2, electron density was observed corresponding to the CshB_NR2 polypeptide chain beyond the final β‐strand of the protein (β14). This density corresponds to an extended loop punctuated by a short α‐helix (α6), which traverses the connecting loops between β6/β7, β8/β9, β10/β11, and β12/β13, and occupies a groove formed by the packing of α1 against the larger of the two β‐sheets.

### 3.3 Reduced Fn‐binding affinity in CshB_NR2 correlates with a loss of electrostatic charge in the ligand‐binding site

**FIGURE 4:**
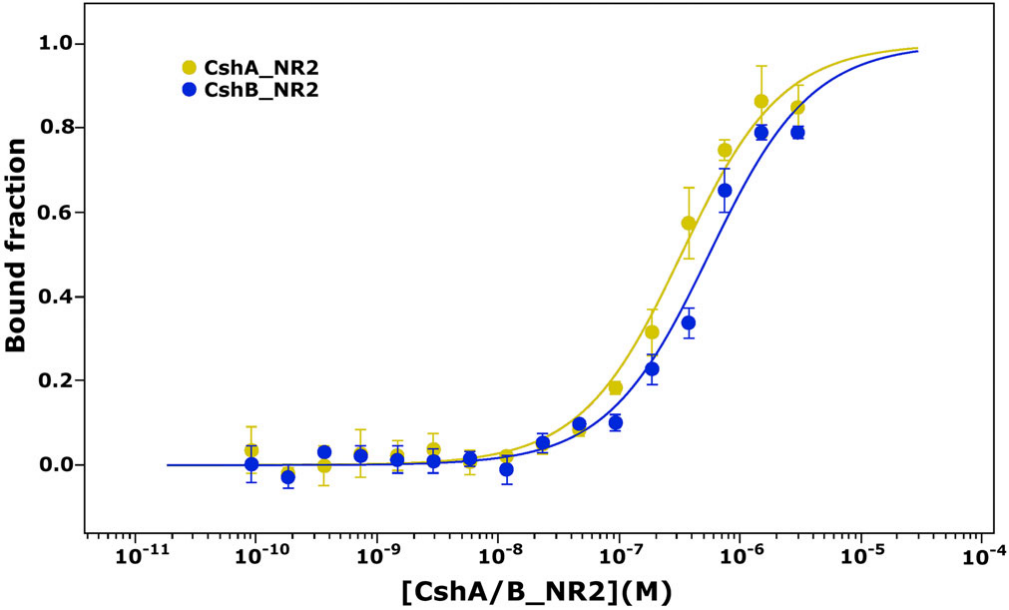
In vitro quantitation of cFn binding by CshA_NR2 and CshB_NR2 polypeptides. MST traces of cFn binding by CshA_NR2 (yellow) and CshB_NR2 (blue). All experiments were conducted using cFn at a final concentration of 50 nM. Data have been fitted to a single‐site dose–response binding model. Error bars correspond to standard deviations from the mean calculated from three repeats of each measurement.

Having confirmed the identity of the Fn‐binding pocket of CshA_NR2, we next sought to investigate the Fn‐binding properties of CshB_NR2. Inspection of the CshB_NR2 structure reveals the presence of an equivalent binding pocket, however, the amino acid composition of this region differs from that seen in CshA_NR2 (Figure 3C). The CshB_NR2‐binding pocket is appreciably less negatively charged, predominantly as a consequence of the substitution of residues Asp300, Asp337, and Asp338 in CshA_NR2 with tyrosine (Tyr288), methionine (Met326) and asparagine (Asn327), which occupy equivalent positions in CshB_NR2. To investigate the impact of these substitutions on Fn binding, the affinities of both CshA_NR2 and CshB_NR2 for human cFn were experimentally determined using MST. To ensure equivalency, purified recombinant CshB_NR2 generated using *csh*B_NR2::pOPINF, which encodes an N‐terminally hexa‐histidine tagged variant of CshB_NR2, was employed for these studies. From these analyses, *K*_D_ values for cFn binding by CshA_NR2 and CshB_NR2 were calculated to be 302 ± 44 and 530 ± 105 nM, respectively (Figure 4). These data equate to an ~1.7‐fold reduction in cFn affinity in CshB_NR2 as compared to CshA_NR2, and are in agreement with previous gene knockout experiments, where a Δ*csh*A strain of *S. gordonii* was shown to exhibit a greater degree of impairment in Fn binding as compared to a Δ*csh*B strain.^10^

### 3.4 CshA/B_NR2 homologues vary in both ligand‐binding pocket composition and capping loop sequence

**FIGURE 5:**
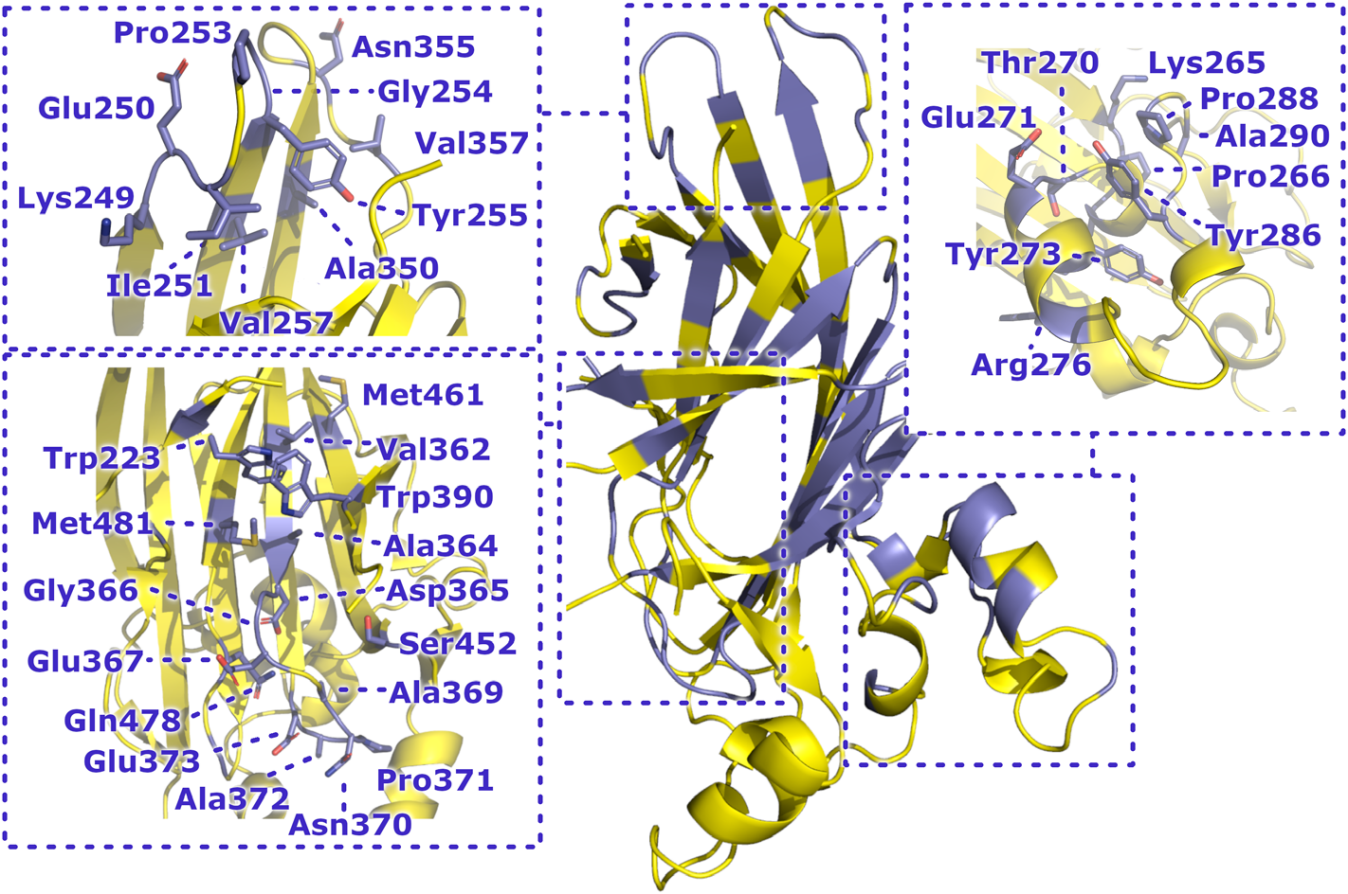
Residue conservation in CshA/B_NR2 homologues. Identified residues have been mapped onto the structure of monomeric CshA_NR2. Residues with a percentage conservation of ≥95% across all 500 sequences analyzed are colored dark blue.

Due to the observed divergence in Fn‐binding pocket composition in CshA_NR2 as compared to CshB_NR2, we next undertook a bioinformatic conservation analysis in an effort to identify regions within CshA/B_NR2 homologues that exhibit high levels of sequence divergence. To generate a suitable sample size of representative homologues with sufficient sequence diversity, a search using the PSI‐BLAST server was conducted.^25^, ^26^ This returned 500 CshA/B_NR2 homologues from *Streptococcacae*, with CshA_NR2 sequence identities ranging from 40% to 99%. Returned sequences had >90% CshA/B_NR2 sequence coverage and *E* scores of <0.005, ensuring that the sample set comprised bona fide CshA/B_NR2 homologues.^46^ Sequences were aligned and conserved residues (>95% conservation) mapped onto the CshA_NR2 structure (Figure 5). This analysis identified several regions of high sequence conservation in CshA_NR2, CshB_NR2 and their relatives. These include the hydrophobic interface between the two central β‐sheets and the connecting regions between β2/β3, β3/β4, β6/β7, β7/β8, and β9/β10. Aligned sequences of the identified homologues were visualized in CLC Genomics Workbench 12 and revealed frequent amino acid insertions in strands β1 and β4, and in the regions that correspond to the interface between β3/β4 and β9/β10 (Figure S2). Notably, significant sequence variability was observed in the β11/β12 loop, which caps the Fn‐binding pocket, and in the Fn‐binding pocket itself, implying that these functionally important regions are highly mutable.

### 3.5 Structural homologues of CshA/B_NR2 constitute a family of highly mutable adhesive domains that bind components of the extracellular matrix

**FIGURE 6:**
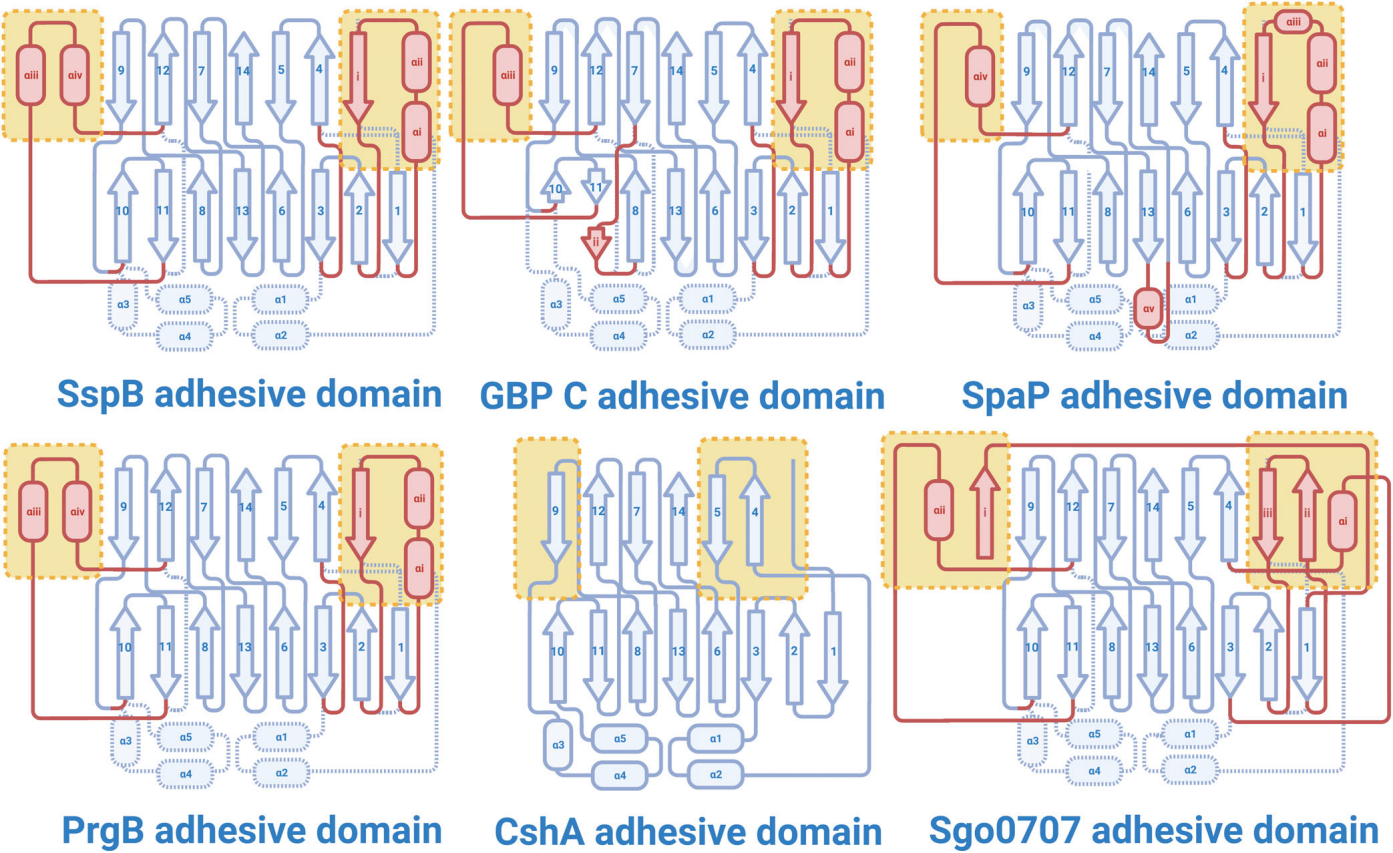
Topology diagrams of CshA/B_NR2 structural homologues. Arrows denote β‐strands, rectangles denote α‐helices and lines represent loops. The conserved β‐sandwich core of each fold is colored dark blue. Structural elements colored light blue (with dotted outlines) are those that are absent from the fold relative to CshA/B_NR2, those colored red are present in the fold relative to CshA/B_NR2. β‐strands are numbered relative to the CshA_NR2 model, with additional elements labeled with roman numerals. Structural elements that constitute capping loops of the ligand‐binding pocket of each of the domains shown are highlighted in yellow boxes.

**FIGURE 7:**
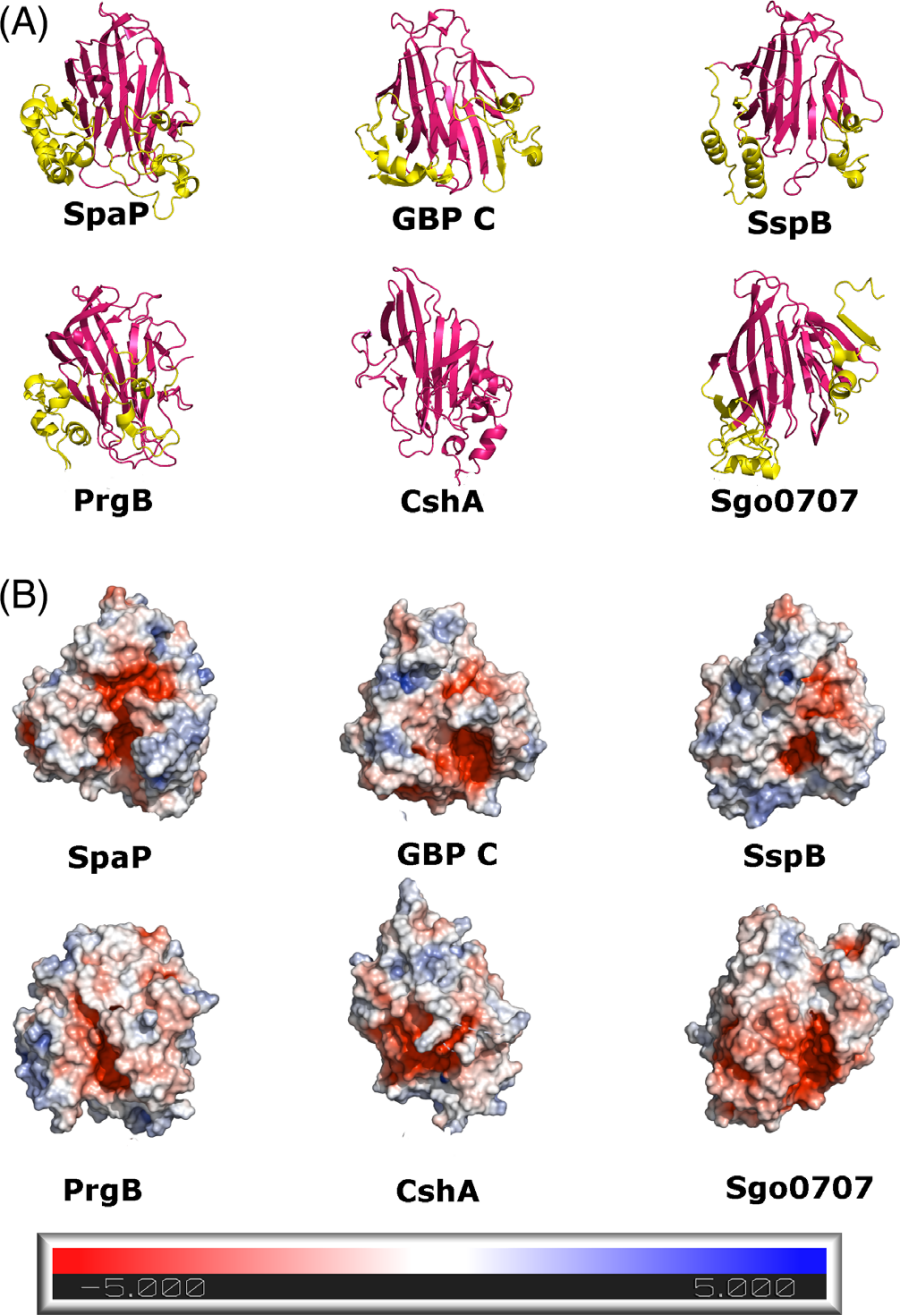
Comparative folds of CshA/B_NR2 structural homologues. (A) Ribbon representations of CshA/B_NR2 structural homologues, with the conserved core of each protein colored pink, and accessory secondary structural elements in yellow. (B) Electrostatic surface potentials of CshA/B_NR2 structural homologues. The Poisson–Boltzmann electrostatic scale bar shows potentials of ± 5 kT/e (red to blue, respectively).

Having identified the potential for sequence variability in the Fn‐binding pocket and associated capping loop of CshA/B_NR2 homologues, we next sought to investigate the degree of structural variability tolerable in these regions. Highly mutable folds are often recalcitrant to identification using sequence‐based methods, as distant homologues may be sufficiently divergent in their sequences to preclude detection.^47^ In such instances, more distantly related polypeptides may only be identifiable using searches based on structural rather than sequence identity.^48^ To address this, we performed DALI searches using the structures of monomeric CshA_NR2 and CshB_NR2 as search models. Of the identified structural homologues, many were found to be adhesive domains resident within fibrillar adhesins. Surprisingly, of those domains which had been subjected to experimental characterization, each had been shown to bind partner ligands within the extracellular matrix (ECM). The highest scoring hits identified (*Z*‐scores = 8.4–6.7, RMSD = 3.3–3.1 Å) were the variable domains of SpaP, SspB, and GbpC, the previously highlighted N1 domain of Sgo0707,^8^ and the adhesive domain of PrgB. Lower scoring candidates (*Z*‐scores = 5.3, 4.3, 4.0, RMSD = 4.1–2.8 Å) included the N1 subdomain of Epf, the CnaA‐like domain of GspB and the KRT10‐binding region of PsrP. Despite possessing low sequence identity to CshA/B_NR2 (Table S2), each of the highest‐scoring structural homologues retains the same core β‐sandwich fold as that observed in both CshA_NR2 and CshB_NR2.^7^, ^49^, ^50^, ^51^, ^52^ Notably, however, in some instances, this fold is augmented via the acquisition of up to three additional β‐strands (Figures 6 and 7A). All identified structural homologues of CshA/B_NR2 present a sizeable negatively charged pocket on their surface, analogous to the Fn‐binding site of CshA/B_NR2 (Figure 7B). In addition, in each instance access to this site is regulated by a flexible capping loop. Significant variation is seen in the size and amino acid composition of both the binding pocket and capping loop in different NR2 structural homologues, with the former being largely dictated by the region preceding β3, and that which connects β11–β12 (relative to our CshA/B_NR2 structures; Figure 6). The observed structural differences between these domains appear to enable the acquisition or loss of partner binding specificity as a consequence of mutation within the gated binding pocket and/or extension of the core β‐sandwich fold via atrophy and elaboration of the central β‐sheets.^53^ The high structural identity of CshA_NR2, CshB_NR2, and the N1 domain of Sgo0707 to adhesive domains within the recently defined PAD family of bacterial polypeptides^6^ indicates that these three polypeptides should be classified as members of this group.

### 3.6 CshA/B_NR2‐like domains are widely distributed in polypeptides from both Gram‐positive and Gram‐negative bacteria

Having established that CshA/B_NR2 are members of the PAD family of adhesins, we next investigated the breadth of bacterial polypeptides that house homologous CshA/B_NR2 domains. To find the most diverse set of candidate polypeptides, a PSI‐BLAST search was performed, which returned 2056 candidate polypeptides from a range of bacterial species (Table 2). Approximately, 72% of the candidate sequences represent proteins from Terrabacteria (of which 44% originate from *streptococci*), with ~18% coming from Proteobacteria and 9% coming from Bacteroidetes. These data demonstrate the extensive distribution of NR2‐like domains within bacterial polypeptides. Surprisingly, ~28% of the identified homologues were from Gram‐negative species, of which the majority were soil or marine dwelling,^54^, ^55^ with high‐confidence AlphaFold models (pLDDT >90) demonstrating the homology of the NR2‐like fold (Figure S3). Comparative alignment of the CshA/B_NR2‐like domains, coupled with neighbor‐joining analysis,^56^ reveals a diverse distribution of domain sequences that cluster to form resolvable nodes (Figure S4). Inspection of the aligned full‐length sequences (1804 retrievable, 252 rejected) reveals an overall low percentage sequence identity (most exhibit <40%), indicating that the majority of polypeptides that house CshA/B_NR2‐like domains are not formally CshA/B homologues (Figure S5).

**TABLE 2:** Taxa distribution of the 2056 polypeptides containing CshA/B_NR2‐like domains.

Phylum	Predominant class(es)	Percentage of all PSIBLAST hits
Firmicutes		**47.3**
	Bacilli	47.1
Actinobacteria		**24.3**
	Actinomycetia	24.1
Bacteroidetes		**9.2**
	Sphingobacteriales	2.9
	Flavobacteria	3.7
	Chitinophagaceae	2.3
Proteobacteria		**18.4**
	Alphaproteobacteria	15.4
	Gammaproteobacteria	2.5

*Note*: Values in bold correspond to the total distribution of each phyla in the 2056 polypeptides, expressed as a percentage.

### 3.7 CshA/B_NR2‐like domains colocalise upstream of CshA/B_NR3‐like domains

**FIGURE 8:**
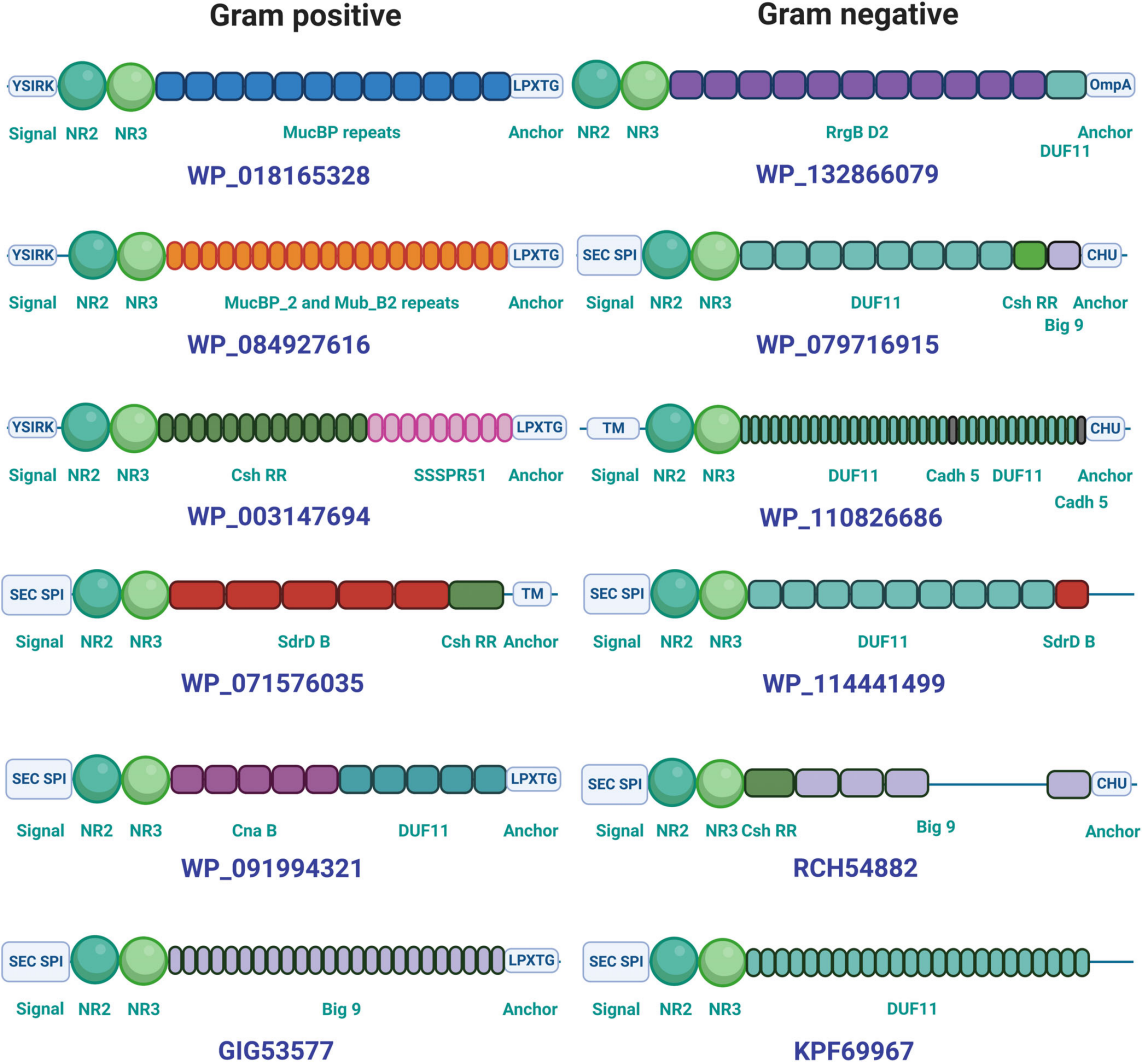
Illustrative examples of various domain architectures in CshA/B_NR2‐like containing polypeptides identified in this study. Examples shown are selected from a pool of 2056 candidate sequences generated during this study. UniProt accession numbers for the proteins shown are provided. Different domain families are depicted in different colors, and are labeled with the name attributed to them in the Interpro database. Domains are not drawn to scale.

Our library of full‐length candidate sequences containing CshA/B_NR2‐like domains next allowed us to investigate the domain architecture of these candidate polypeptides, focusing on a subset of sequences from each node. InterPro annotation of full‐length proteins from these nodes reveals a surprisingly diverse collection of CshA/B_NR2‐like domain‐containing polypeptides with disparate architectures incorporating a diverse array of stalk‐forming domains (Figure S6). This analysis also reveals that CshA/B_NR2‐like domains frequently co‐localize in multi‐domain polypeptides with a C‐terminal partner domain, which exhibits sequence identity to CshA/B_NR3 (24%–36%; Figure 8). Previous studies of Fn binding by CshA revealed that the NR3 domain of this polypeptide is dispensable for Fn binding,^8^ however, the retention of NR3‐like sequences neighboring NR2‐like domains implies an unknown but important function. Closer inspection of the predicted NR3‐like regions within the candidate sequences reveals disparity in the assigned C‐terminal residues of these domains, with many instances of overlap with the N‐terminus of a downstream stalk forming domain, suggesting previous misassignment of the C‐terminal domain boundary within CshA.^8^ To resolve this, we sought to define the primary N‐terminal residues of each downstream stalk domain, using the Needleman–Wunsch alignment algorithm as applied to the appropriate consensus sequence from the Conserved Domain Database (CDD).^57^ These analyses revealed that the N‐termini of stalk domains downstream of CshA/B_NR3 domains occur 12–17 residues downstream of a highly conserved (>88% of sequences) GEVED motif (Figure S7). These findings are consistent with CshA/B_NR3‐like domains being significantly smaller in size than previously proposed. We thus defined this as the “GEVED” domain, which has been subsequently added to the Pfam database with accession number PF20009, and herein reclassifies the NR3 region of CshA/B as comprising a GEVED domain followed by an extended repeat region 1 (RR1) domain. Together, these findings demonstrate that CshA/B_NR2 domains are invariably partnered with a downstream GEVED domain, whose retention indicates an important role in fibrillar adhesins.

### 3.8 CshA/B_NR2 domains are localized to the tips of adhesins

**FIGURE 9:**
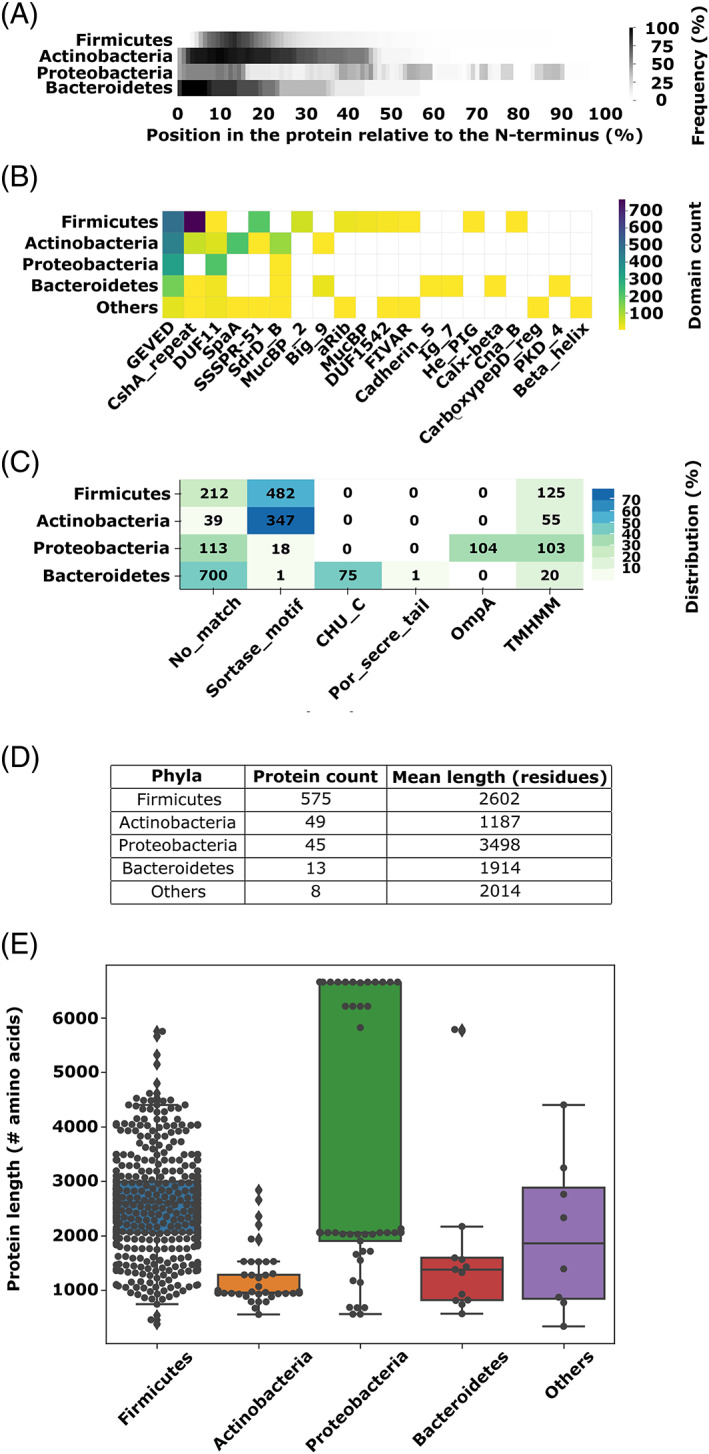
Distribution of CshA/B_NR2‐like domains and their full‐length proteins. (A) Locus of CshA/B_NR2‐like domains in full‐length polypeptides, separated by phyla (690 sequences). (B) Plot of length of full‐length polypeptides identified in the PSI‐BLAST sample pool (690 sequences). (C) Frequency of domain occurrence in polypeptides containing CshA/B_NR2‐like domains categorized by phyla (2056 sequences). (D) Frequency of signal peptide and anchor domains in polypeptides categorized by phyla (2056 sequences). (E) Total full‐length polypeptide count and average length categorized by phyla (690 sequences).

Having assessed the breadth of sequences that contain CshA/B_NR2‐like domains, we next sought to explore their relationship with their partner stalk domains. Our bioinformatic analyses demonstrated that CshA/B_NR2‐like sequences are found within a diverse range of polypeptides that incorporate a wide variety of repetitive stalk domains. These findings are consistent with CshA/B_NR2‐like domain‐containing proteins being extracellular polypeptides that confer adhesive functions. Of the 2056 candidate sequences, we identified as containing a CshA/B_NR2‐like domain, 690 were classified as putative adhesins based on the presence of repeating stalk domains, along with an anchoring motif at their C‐terminus. The majority of these 690 sequences represent bacterial polypeptides with unassigned cryptic functions (Figure 9). Our analysis reveals that CshA/B_NR2‐like domains and CshA/B_GEVED‐like domains predominantly reside at the tip of repetitive multi‐domain stalk proteins (Figure 9A). Hidden Markov model (HMM) characterization of domains within each polypeptide was used to assess domain organization within full‐length sequences, revealing a diverse set of 24 domains that frequently partner with CshA/B_NR2‐like and CshA/B_NR3‐like sequences (Figure 9B,C).

### 3.9 Domain shuffling drives adhesin diversification

As our adhesin library represents proteins found in a variety of both Gram‐positive and Gram‐negative species (Figure 9D), we next investigated the primary differences in domain organization within these polypeptides. Significantly, each of our 690 candidate sequences possesses a C‐terminal cell surface anchoring domain or motif, consistent with an extracellular function. In Gram‐positive species, this is universally a sortase‐specific (most commonly LPXTG) motif required for cell‐wall attachment, whereas in Gram‐negative species, five cell surface anchoring motifs were identified including OmpA‐like domains (predominantly in Proteobacteria) and CHU_C homologues (Bacteroidetes) (Figure 9C). Our analyses also reveal that protein length directly correlates with bacterial phyla. For example, sequences housing CshA/B_NR2‐like domains from Firmicutes are significantly longer than those from Actinobacteria and Bacteroidetes, while Protobacteria encode two distinct populations of CshA/B_NR2‐like domain containing polypeptides that can be readily distinguished based on sequence length (Figure 9D,E). In addition, CshA/B_NR2‐like domains are found partnered with a diverse array of stalk‐forming domains whose frequency of use is phyla‐dependent (Figure 9B). In Firmicutes, CshA/B_NR2‐like domain containing polypeptides predominantly incorporate CshA/B‐like, SSSPR51‐like, and MucBP_2‐like stalk‐forming repeat domains, whereas CshA‐like, SpaA‐like, and SdrD_B‐like repeat domains predominate in Actinobacteria, with Proteobacteria most frequently employing DUF11 repeats.

## 4 DISCUSSION

In previous studies, we investigated the molecular basis of Fn binding by the *S. gordonii* fibrillar adhesin CshA and employed an integrative structural biology approach to establish the molecular architecture of this important polypeptide.^8^, ^58^ Here, we extend the scope of our studies of Csh adhesins by focusing on the CshA paralogue CshB; investigations which have directed us to undertake a comprehensive analysis of the distribution of CshA/B_NR2‐like domains in bacteria.

We have elucidated the X‐ray crystal structure of a strand‐swapped homodimer of the Fn‐binding NR2 domain of CshB and used this information to produce a molecular model of the biologically relevant monomeric form of this polypeptide. CshB_NR2 possesses a distinctive β‐sandwich core fold analogous to that of CshA_NR2, with divergence in the amino acid composition of the surface exposed Fn‐binding cleft. The most significant difference between the Fn‐binding sites of these two proteins is their local electrostatic charge, which is significantly reduced in CshB_NR2 as compared to CshA_NR2. Complementary binding studies demonstrate that these changes translate into a reduction in Fn‐binding affinity in vitro, consistent with data from previous gene knockout studies in *S. gordonii*.^10^ This loss of Fn affinity could represent the early stages of domain neofunctionalisation, wherein the mutations acquired by a gene‐duplicated paralogue are tolerated, thus enabling the acquisition of a new function, such as a change in substrate specificity.

In studies focused on the identification of structural homologues of CshA/B, we have identified a number of structurally related adhesive domains from other well‐characterized adhesins. Notably, these structural homologues exhibit low sequence identity (13%–15%) to both CshA_NR2 and CshB_NR2, and include members of the recently defined PAD family.^6^ Each of the identified structural homologues resides in an adhesin known to bind an ECM substrate (Table 3), though significantly none have been shown to selectively target cFn. The observed divergence in partner ligand‐binding specificity correlates with observable structural changes in the ligand‐binding pocket of each CshA/B_NR2‐like homologue. These findings are consistent with our conservation analysis, which indicates that indel mutations are prevalent in the ligand‐binding site and associated with the capping loop of CshA/B_NR2‐like domains. Each of the identified domains retains a negative electrostatic charge within its binding pocket, implying that this is a requirement for substrate binding. Based on our findings, we propose that both CshA/B_NR2 and Sgo0707_N1 be classified as PAD family members.

**TABLE 3:** Example PADs and their respective target molecules.

PAD	PDB ID	Target ECM substrate of PAD	Reference
CshA NR2	5l2D	Fibronectin	^8^
CshB NR2	6YZG	Fibronectin	This study
SspB V domain	2WD6	Polysaccharides	^50^
SpaP V domain	3IOX	Unknown	N/A
GbpC V domain	6CAM	Dextran	^59^
GbpC V domain	6CAM	gp340	^51^
PrgB adhesin domain	6GED	DNA	^52^
Sgo0707 N1	4IGB	Collagen (Type I)	^7^

Complementary bioinformatic techniques have also been employed to investigate the prevalence of CshA/B_NR2 homologues in non‐Csh polypeptides. We have identified a wide‐spread distribution of CshA/B_NR2 homologues in functionally cryptic bacterial extracellular polypeptides, which based on our findings, we predict with high probability to function as adhesins. Analysis of the domain organization of these sequences reveals that CshA/B_NR2‐like domains invariably colocalise upstream of CshA/B_GEVED‐like domains, indicating an important role for the latter in protein structure and/or function.

Our HMM studies indicate that domain shuffling acts as a primary driver of adhesin assembly. This process affords a mechanism by which bacteria can generate novel adhesins by integrating distinct domains, tailored to bind to specific targets, with appropriate partner stalk sequences, yielding a panoply of mosaic architectures. Whether the adhesive capabilities of these polypeptides originate solely from the CshA/B_NR2/GEVED‐like domains is unclear, but the acquisition of different domains and their preferred usage by specific bacterial phyla suggests a species selective pressure.

## AUTHOR CONTRIBUTIONS

**Rob Barringer:** Conceptualization; investigation; formal analysis; writing – original draft; writing – review and editing; methodology; visualization. **Alice E. Parnell:** Investigation; formal analysis; writing – original draft; writing – review and editing; methodology. **Aleix Lafita:** Investigation; formal analysis; writing – review and editing; methodology. **Vivian Monzon:** Investigation; formal analysis; writing – review and editing; methodology. **Catherine R. Back:** Formal analysis; writing – review and editing; methodology; validation. **Mariusz Madej:** Investigation; formal analysis; writing – review and editing; methodology. **Jan Potempa:** Formal analysis; writing – review and editing; resources; supervision. **Angela H. Nobbs:** Conceptualization; formal analysis; writing – review and editing; supervision; methodology; resources. **Steven G. Burston:** Conceptualization; formal analysis; writing – review and editing; supervision; methodology. **Alex Bateman:** Conceptualization; formal analysis; writing – original draft; writing – review and editing; supervision; methodology; data curation; project administration; resources; validation. **Paul R. Race:** Formal analysis; writing – original draft; writing – review and editing; funding acquisition; supervision; methodology; data curation; project administration; resources; validation; conceptualization.

## FUNDING INFORMATION

This study was funded by BBSRC grants BB/L01386X/1, BB/M012107/1 and BB/T001968/1, and through the award of a BBSRC SWBio Ph.D studentship to RB (BB/T008741/1). AL, AB and VM are supported by European Molecular Biology Laboratory core funds.

## CONFLICT OF INTEREST STATEMENT

The authors report no conflicts of interest.

### PEER REVIEW

The peer review history for this article is available at https://www.webofscience.com/api/gateway/wos/peer-review/10.1002/prot.26487.

## Supporting information


**Data S1:** Supporting Information

## Data Availability

The X‐ray crystal structure of CshB_NR2 has been deposited in the PDB with the accession code 6YZG. The data that support the findings of this study are available as supplementary files.
