# Supplementary material for: Domain shuffling of a highly mutable ligand‐binding fold drives adhesin generation across the bacterial kingdom
Source: Proteins. 2023 Mar 20;91(8):1007–20. doi: 10.1002/prot.26487 (PMC10952558; doi:10.1002/prot.26487)
Supplement: Supplementary file 1 — Data S1: Supporting Information [file PROT-91-1007-s001.pdf]

Supplementary Tables and Figures

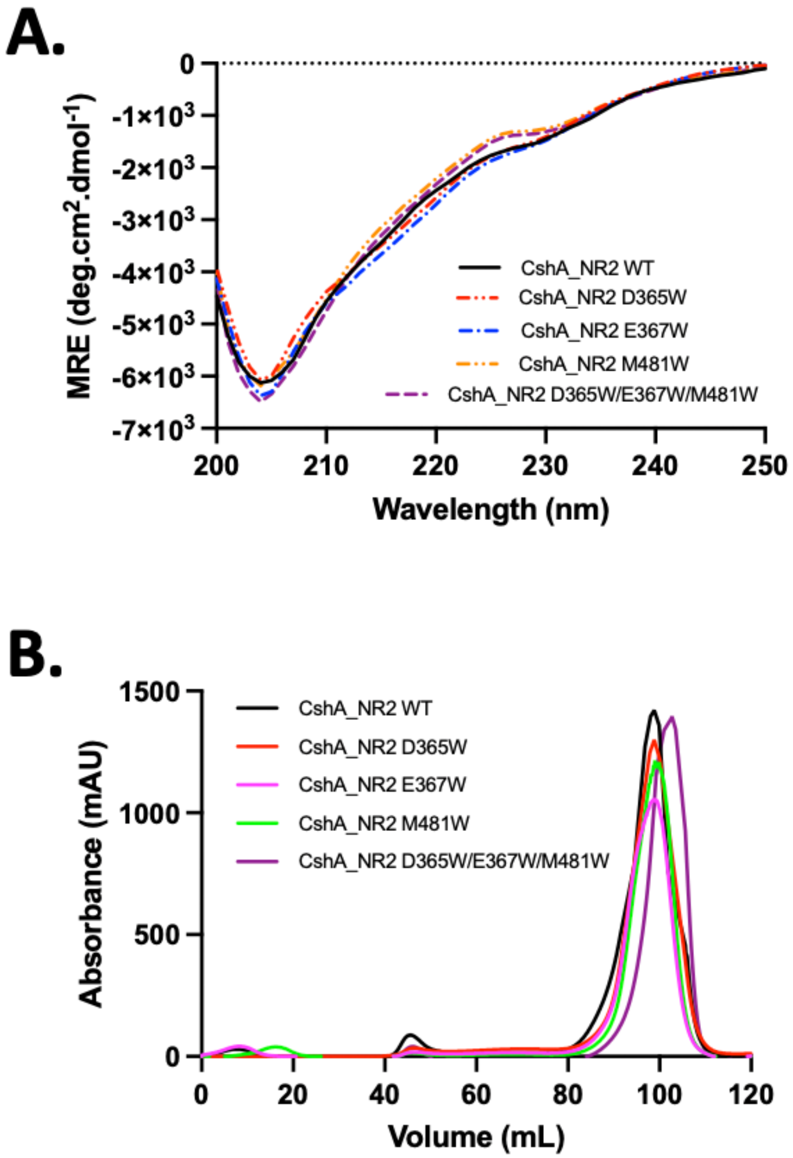

Figure S1. Biophysical characterisation of monomeric CshA_NR2 polypeptides. A) Far UV circular dichroism (CD) spectra of monomeric CshA_NR2 WT (black), CshA_NR2 D365W (red), CshA_NR2 E367W (blue), CshA_NR2 M481W (orange), and CshA_NR2 D365W/E367W/M481W (purple). CD spectra were collected from samples in 10 mM Sodium Phosphate, 100 mM Sodium Fluoride, pH 7.4, at 4°C. B) Size exclusion chromatograms of CshA_NR2 WT (black), CshA_NR2 D365W (red), CshA_NR2 E367W (pink), CshA_NR2 M481W (green), and CshA_NR2 D365W/E367W/M481W (purple). All data were collected using a Hi-Load 16/600 Superdex 75 column (GE Healthcare) pre-equilibrated in 637 mM NaCl, 2.7 mM KCl, 10 mM Na_2HPO_4, 1.8 mM KH_2PO_4, pH 7.4. The absorbance of the column eluant was monitored at 280 nm throughout.

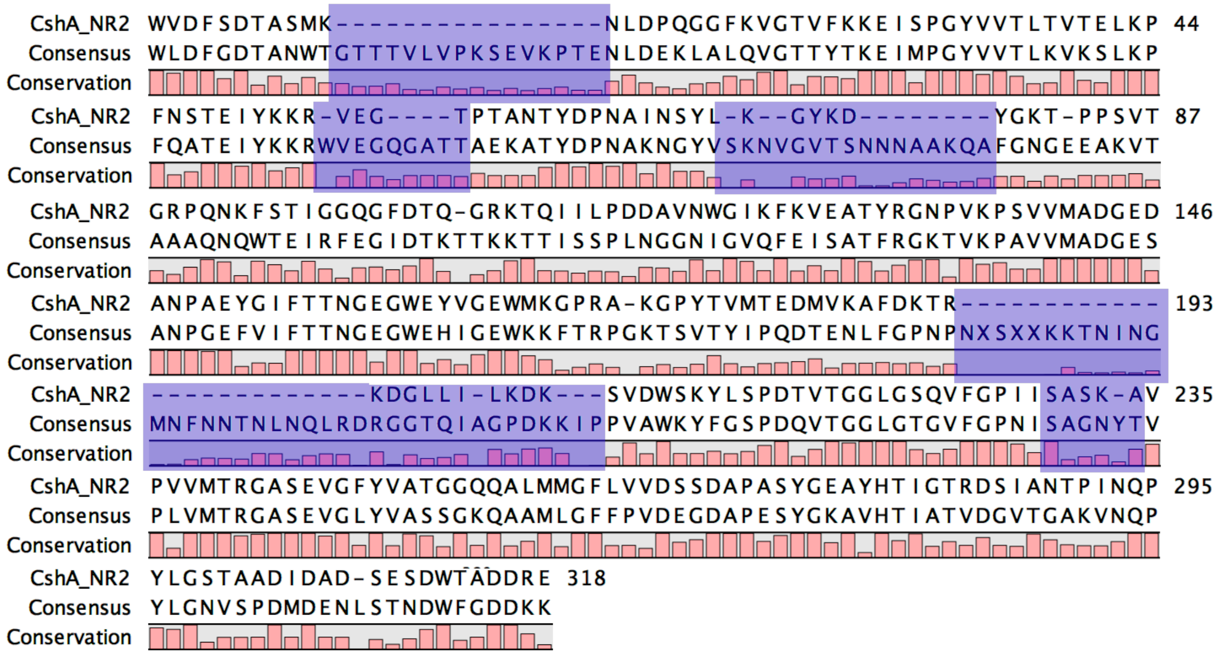

Figure S2. Conservation of CshA_NR2-like domains as judged from the MSA of 500 closely related CshA_NR2 homologues, with indel regions labelled. Indel regions correspond to β1, the loop between β3/β4, β4, the loop between β9/β10, and the loop between β11/β12, respectively. Conservation of the consensus sequence is indicated using a bar chart, with values ranging from 0% to 100%. Positions without a majority sequence are represented in the consensus sequence by 'X'.

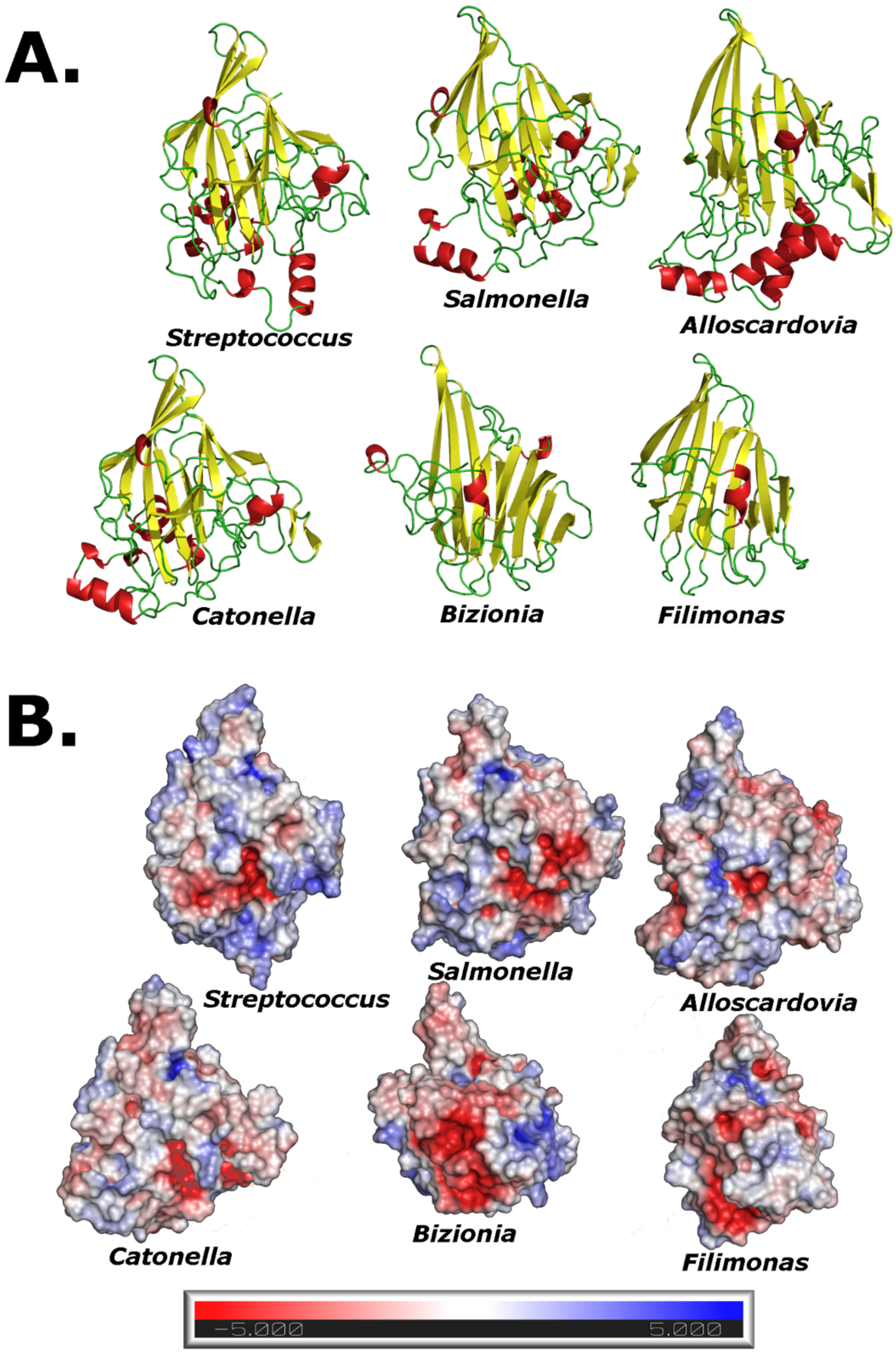

Figure S3. AlphaFold predictions of NR2-like domains from Gram-negative bacteria. A) Cartoon representations of predicted structures from the indentified bacteria are shown, along with an AlphaFold model of CshA_NR2. B) Electrostatic surface potential representation of the structures shown in (A), alongside a Poisson-Boltzmann electrostatic scale bar showing a potential of -/+ 5 kT/e (red to blue). Uniprot accessions of the identified NR2-like containing sequences are: *Salmonella* - A0A1S0Z4E8; *Alloscardovia* - A0A1Y2SYH1; *Catonella* - RKW15100; *Bizionia* - G2EA05; *Filimonas* - A0A2U1A2U3. Average pLDDT scores from Alphafold were >90%.

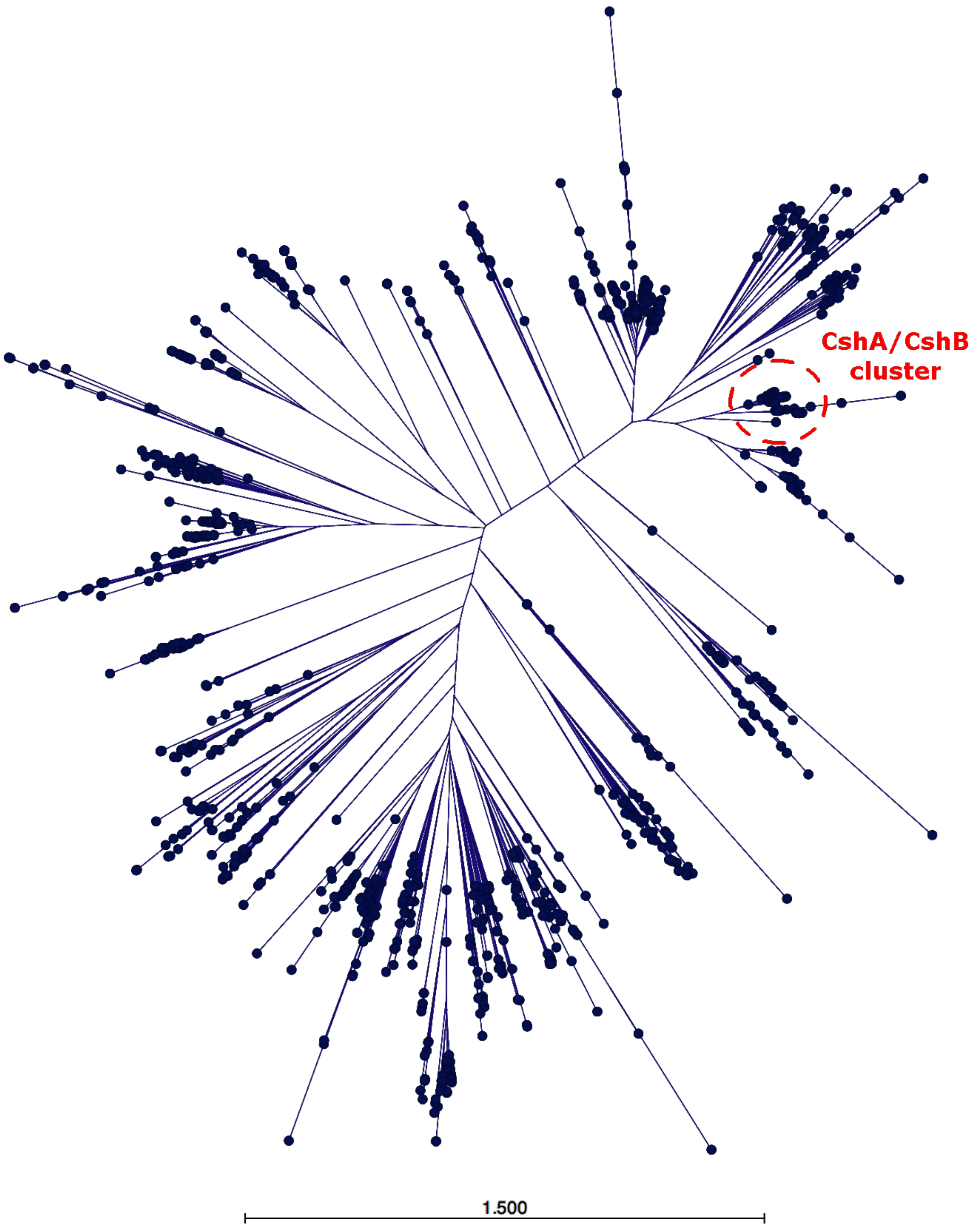

Figure S4. A radial representation of the NJ tree contructed from 2056 diverse CshA_NR2-like homologues. Clusters containing CshA_NR2 and CshB_NR2 are indicated. The scale bar represents average substitutions per site.

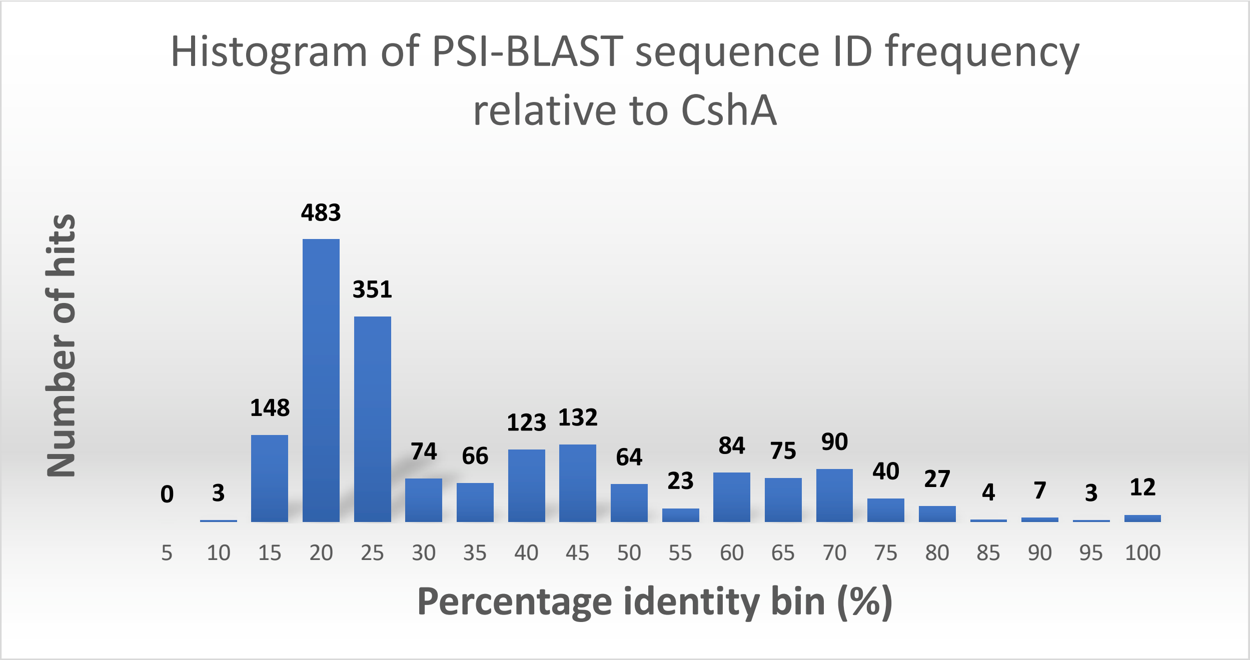

Figure S5. Histogram showing the frequency of the 1804 full-length sample pool sequence identities relative to CshA. Data are sorted into identity bins in 5% increments relative to CshA. Numbers on the horizontal axis show the upper boundary of each bin, numbers above the bars show the count of sequences belonging to each bin.

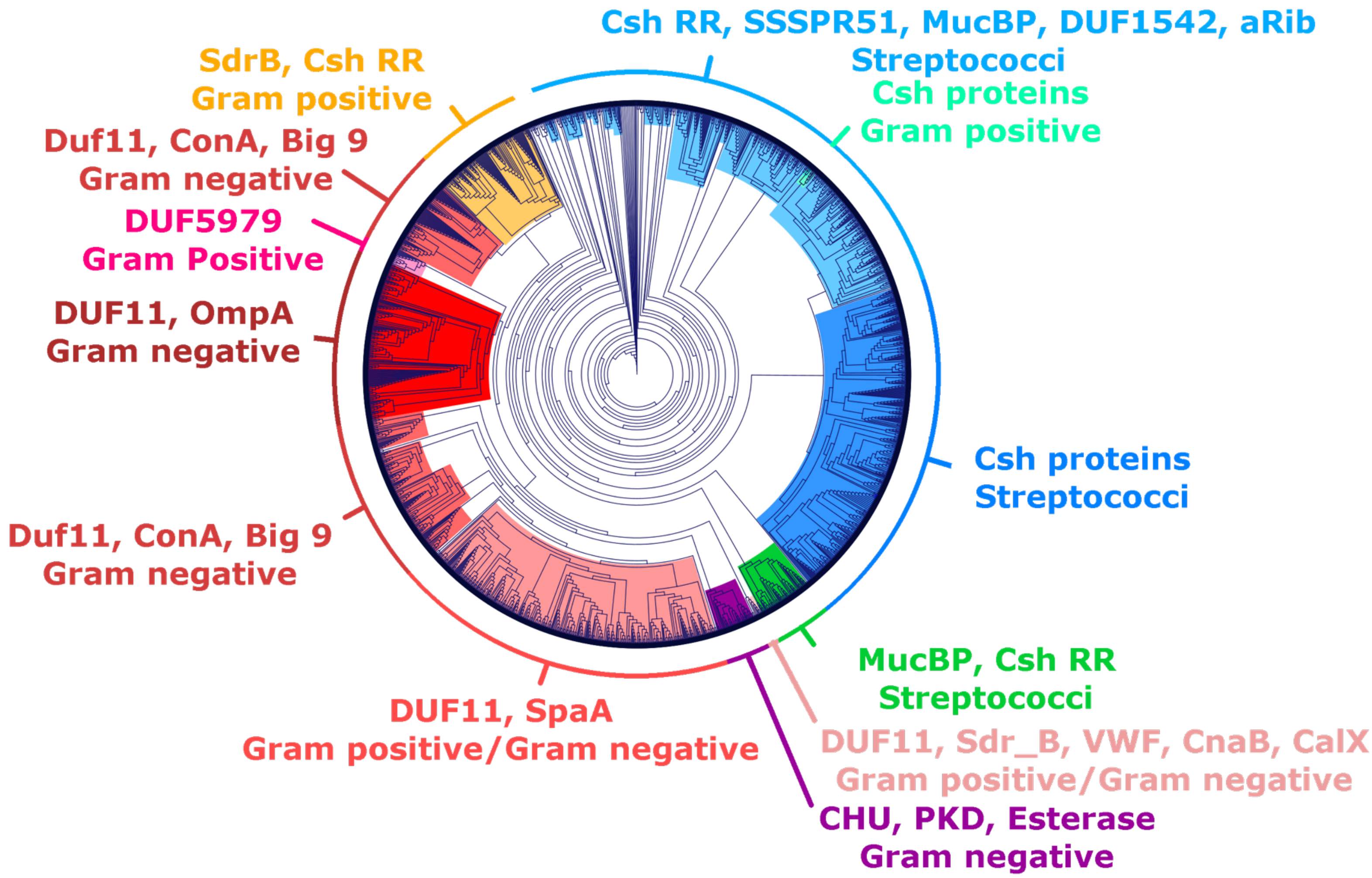

Figure S6. Circular cladogram of NJ tree constructed from 2056 diverse CshA_NR2-like homologues. Full-length sequence architectures were characterised using Interpro. Clusters showing similar architectures are colour coded, denoting the domains that are commonly presented alongside CshA/B_NR2 in each cluster.

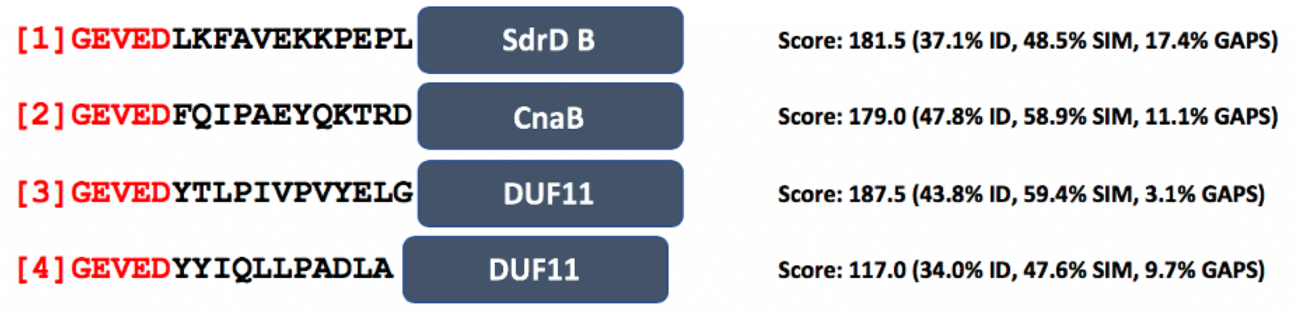

Figure S7. Loci of domains positioned downstream of the CshA_NR3 region (so-called 'Annex sites'). Proteins are from architectures shown in Figure 8, where the downstream domains had an Emboss Needle score of >100. Annotated blocks denote specified domains with the conserved GEVED motif highlighted in red and the subsequent residues before the downstream domain shown in black. Emboss Needle alignment statistics are given for each downstream domain. Bracketed numbers denote the following Uniprot accessions: [1] WP_071576035.1 [2] WP_091994321.1 [3] WP_110826686.1 [4] WP_114441499.1

Strain, plasmid or primer	Relevant genotype, phenotype or sequence	Source
S. gordonii DL1 – Challis strain	Wild type	60
pOPINF	Protein expression vector, incorporating an N-term MAHHHHHHSSGLEVLFQGP tag (AmpR)	13
pOPINE	Protein expression vector, incorporating a C-term KHHHHHH tag (AmpR)	13
CshB_NR2 forward primer pOPINF	**<u>AAGTTCTGTTTCAGGGCCCG</u>**TGGCTTGATTTTTCAGATAG	N/A
CshB_NR2 reverse primer pOPINF	**<u>ATGGTCTAGAAAGCTTTA</u>**CTTTTTATCATCCAAAACCCAG	N/A
CshB_NR2 forward primer pOPINE	**<u>AGGAGATATACCATG</u>**TGGCTTGATTTTTCAGATAG	N/A
CshB_NR2 reverse primer pOPINE	**<u>GTGATGGTGATGTTT</u>**CTTTTTATCATCCAAAACCCAG	N/A

Table S1. Source of wild type bacteria, expression vectors, and primers used in this study. Vector-complementary sequences within pOPIN plasmids are underlined in bold.

	CshA	PrgB	GbpC	SspB	SpaP
**Sgo0707**	13.17	14.45	14.17	13.19	10.16
**CshA**		16.20	15.35	13.61	17.98
**PrgB**			26.34	27.50	20.58
**GbpC**				29.07	29.84
**SspB**					34.85

Table S2. Sequence identity matrix of adhesive domains. Percentage of sequence identity after MUSCLE alignment using the EMBL-EBI server is depicted.
